# What Plasma Can Tell Us When Tissue Cannot: A Case Report of Genomic Testing in mCRPC and Clinical Response to Treatment With the PARP Inhibitor Rucaparib 

**DOI:** 10.3389/fonc.2022.951348

**Published:** 2022-08-01

**Authors:** Daniel P. Petrylak, Simon P. Watkins, Andrea Loehr

**Affiliations:** ^1^ Smilow Cancer Center, Yale School of Medicine, New Haven, CT, United States; ^2^ Clinical Science, Clovis Oncology UK, Ltd, Cambridge, United Kingdom; ^3^ Translational Medicine, Clovis Oncology, Inc., Boulder, CO, United States

**Keywords:** plasma, BRCA, prostate cancer, PARP inhibitor, case report, poly(ADP-ribose) polymerase

## Abstract

**Background:**

The poly(ADP-ribose) polymerase (PARP) inhibitor rucaparib was approved in the United States based on the phase 2 TRITON2 study of patients with *BRCA1* or *BRCA2* (BRCA)–mutated metastatic castration-resistant prostate cancer (mCRPC). Although genomic screening is recommended as part of a comprehensive assessment of prostate cancer prognosis and treatment options, the best way to select patients with mCRPC for treatment with a PARP inhibitor depends on individual clinical circumstances. For example, assessment of tumor tissue may not always be feasible. Genomic testing of DNA from plasma has become more readily available, providing a minimally invasive option to evaluate DNA from primary and metastatic lesions simultaneously.

**Case Presentation:**

A patient from TRITON2 with BRCA-mutated mCRPC had a response to the PARP inhibitor rucaparib and remained on treatment for 32 weeks, which was >2 times longer than the duration of each of his prior therapies (bicalutamide, docetaxel, abiraterone). The patient enrolled in TRITON2 based on results of local genomic testing of an archival biopsy that indicated the presence of a *BRCA1* T1399I (allelic fraction, 19%) mutation. Local testing also identified an *ATM* G1663C mutation, a *TP53* P191del mutation, and a *BRAF* K601E mutation. Analysis of a plasma sample obtained before the patient started rucaparib detected the same alterations as those in the archival biopsy, but it also revealed the presence of a *BRCA2* homozygous loss (whole gene, 26 of 26 exons) and several other alterations of unknown functional impact. We hypothesize the response of the patient’s tumor to rucaparib was likely driven by DNA damage repair deficiency caused by homozygous loss of all *BRCA2* exons. Following discontinuation from rucaparib due to clinical disease progression, the patient received carboplatin and cabazitaxel for ≈3 weeks. The patient died due to progression of his disease.

**Conclusions:**

A notable aspect of this case is the differences in alterations detected in the archival tumor sample and a more recent plasma sample. This highlights the advantages of plasma testing compared with tissue testing when selecting targeted therapies for treatment of mCRPC; however, physicians must determine which tool presents the best solution for each individual case.

## Introduction

Molecular characterization of prostate cancer has become increasingly relevant with the identification of high-risk hereditary factors (1–4) and development of targeted therapies with genomic biomarkers. Among the targeted therapies approved for patients with metastatic castration-resistant prostate cancer (mCRPC) are the poly(ADP-ribose) polymerase (PARP) inhibitors olaparib and rucaparib (5, 6), which have demonstrated efficacy in patients with DNA damage repair (DDR) defects, particularly *BRCA1* or *BRCA2* (BRCA) alterations (7, 8). Such mutations are associated with adverse clinical features and poor outcomes in patients (9). Rucaparib was approved in the United States based on the phase 2 TRITON2 study of patients with BRCA-mutated mCRPC (5, 7).

Genomic screening for pathogenic alterations in multiple genes, including *BRCA1* and *BRCA2*, is recommended as part of a comprehensive assessment of prostate cancer prognosis and treatment options (10–13). However, the best way to select patients with mCRPC for treatment with a PARP inhibitor depends on a patient’s individual clinical circumstances. Molecular assessment of tumor tissue, historically the gold standard, may not always be feasible due to lack of tissue samples of sufficient quality or difficulty in obtaining contemporaneous biopsies (14). Genomic testing of cell-free DNA (cfDNA) in plasma has advanced technically and has become more readily available, providing a minimally invasive option to evaluate DNA from primary and metastatic lesions simultaneously (15). Plasma-based assays, such as FoundationOne Liquid CDx (Foundation Medicine, Inc., Cambridge, MA), have been approved as companion diagnostics for the selection of patients for treatment with PARP inhibitors.

In TRITON2, patients were prospectively selected based on alteration status from central tissue, central plasma, or local test results (blood, tissue, and/or plasma), reflecting the real-world landscape of clinical genomic testing in patients with mCRPC. Here we report a case study of a patient enrolled in TRITON2 based on next-generation sequencing (NGS) of an archival biopsy with subsequent investigation of pretreatment plasma cfDNA that revealed additional alterations of interest which we believe to have contributed to the patient’s clinical response to rucaparib.

## Case Presentation

A 52-year-old White man presented with intermittent constipation and back pain, with a history of a decrease in lymphocyte count (documented two months prior). The patient was a never smoker and had no family history of cancer or other cancer risk factors. Initial computed tomography scans revealed stage T4 prostatic adenocarcinoma with invasion into adjacent structures, metastasis to regional lymph nodes (stage N1), and metastases to the liver, bone, and a distant lymph node (stage M1) (16, 17). A retroperitoneal lymph node was biopsied to confirm histology. His prostate-specific antigen (PSA) level was 1291ng/mL.

**Figure 1:**
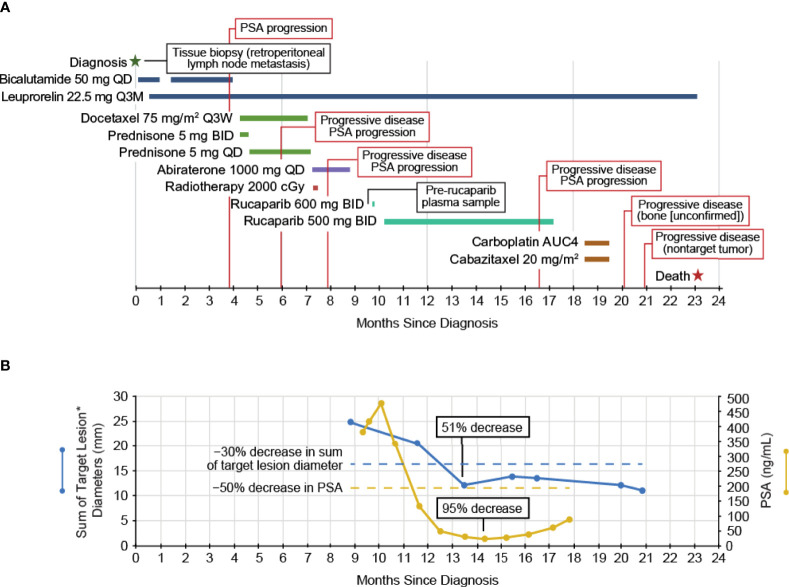
**(A)** The clinical course of the patient and **(B)** the PSA and tumor measurements. *Two measurable liver lesions. BID, twice daily; PSA, prostate-specific antigen; Q3M, every 3 months; Q3W, every 3 weeks; QD, once daily.

The patient started the antiandrogen bicalutamide (oral) shortly after confirmed diagnosis (**Figure 1A**) and a gonadotrophin-releasing hormone agonist, leuprorelin (depot injection), was subsequently initiated to affect androgen deprivation. The patient received treatment until PSA values began to rise ≈15 weeks later and the patient discontinued bicalutamide. Docetaxel (intravenous infusion; 4 cycles) plus prednisone (oral; continuous dosing) was administered as standard of care; prednisone was continued for 1 week after the end of docetaxel treatment for symptom control. The patient ultimately discontinued docetaxel/prednisone due to radiographic disease progression and PSA progression and immediately started on abiraterone as an androgen receptor targeting therapy, which continued for 7 weeks until radiographic disease progression and PSA progression. The patient also received palliative radiation of the right femur and acetabula around the time abiraterone was initiated.

After discontinuing abiraterone, the patient was enrolled in the TRITON2 study based on results of local genomic testing of an archival tissue biopsy (retroperitoneal lymph node metastasis, 90% tumor purity) obtained at initial diagnosis. Local testing utilized the Oncomine™ Comprehensive Assay v3 (Thermo Fisher Scientific Inc., Waltham, MA, USA), which can detect single-nucleotide variants, copy-number variations, gene fusions, and insertions/deletions in 161 cancer-related genes. This local test indicated the presence of a *BRCA1* T1399I (allelic fraction [AF], 19%) mutation (**Table 1**), a novel variant of uncertain significance within a coiled-coil domain which bioinformatics analyses predicted to have a deleterious effect on the BRCA1-PALB2 interaction. A deleterious or probably damaging *ATM* G1663C mutation, a damaging *TP53* P191del mutation, and an oncogenic, activating *BRAF* K601E mutation were also detected; no gene amplifications or gene fusions were detected. TRITON2 patients provided plasma samples for central genomic analysis prior to starting rucaparib. Analysis of the patient’s pre-rucaparib plasma sample was conducted using the FoundationOne Liquid CDx assay, which analyzes 324 cancer-related genes and identifies the same classes of BRCA alterations, including homozygous deletions. The FoundationOne Liquid CDx assay detected the same alterations as the Oncomine analysis of the archival tissue biopsy but also revealed the presence of a *BRCA2* homozygous loss (whole gene, 26 of 26 exons) and several other alterations of unknown functional impact in a plasma sample with 28% tumor content (**Table 1**).

**Table 1:** Results of molecular diagnostic assays.

Biopsy Source Gene Alteration	AF (%)	Predicted Effects/Pathogenicity per Oncomine Report	ClinVar (18) Clinical Significance
Local genomic testing of tissue^a^			
* BRCA1* T1399I	19	Deleterious^b^; Probably damaging^c^	Not reported
* ATM* G1663C	34	Deleterious^b^; Probably damaging^c^	Not reported
* TP53* P191del	84	Damaging^d^	Uncertain significance
* BRAF* K601E	34	Oncogenic, activating mutation	Pathogenic
	**AF (%)**	**Functional Impact per FoundationOne**	**ClinVar** (18) **Clinical Significance**
TRITON2 genomic testing of plasma^e^			
* BRCA2* homozygous loss	–	Pathogenic	–
* BRCA1* T1399I	13.06	Unknown significance	Not reported
* ATM* G1663C	26.74	Pathogenic	Not reported
* TP53* P191del	28.61	Pathogenic	Uncertain significance
* BRAF* K601E	25.92	Pathogenic	Pathogenic
* AR* amplification	–	Pathogenic	–
* ALK* C987R	0.21	Unknown significance	Not reported
* DIS3* H734Q	20.19	Unknown significance	Not reported
* DOT1L* T790M	53.29	Unknown significance	Not reported
* FLT1* P1201L	44.57	Unknown significance	Likely benign
* GNA13:HIVEP1* rearrangement	–	Unknown significance	Not reported
* HSD3B1* I79T	2.54	Unknown significance	Not reported
* IRF4* splice site 493-2_493-1ins87	37.87	Unknown significance	Not reported
* LYN* amplification	–	Focal amplification	–
* NBN* amplification	–	Unknown significance	–
* RAD21* amplification	–	Focal amplification	–

^a^Retroperitoneal lymph node metastasis biopsy at diagnosis (Oncomine™ Comprehensive Assay v3). ^b^SIFT bioinformatic tool. ^c^Per Polyphen bioinformatic tool. ^d^Per PROVEAN bioinformatic tool. ^e^Pre-rucaparib plasma sample (FoundationOne Liquid CDx Assay). AF, allelic fraction.

The patient started at the recommended dose of rucaparib, 600 mg twice daily, but the dose was reduced to 500 mg twice daily due to nausea/fatigue, with the patient ultimately receiving rucaparib for 32 weeks (**Figure 1A**). At enrollment into TRITON2, the patient had >21 bone-associated lesions and multiple liver lesions. Treatment with rucaparib resulted in a confirmed partial response per modified Response Evaluation Criteria In Solid Tumors, version 1.1 (51% decrease in liver metastasis target lesion diameters; **Figure 1B**) lasting 13 weeks, ongoing as of the last radiographic assessment before subsequent anti-cancer therapy, resulting in a rPFS of 29 weeks, with no confirmed progression in bone. The patient also had a confirmed PSA response (maximum decrease, 95%; **Figure 1B**) lasting 28 weeks from the first dose of rucaparib. The patient discontinued rucaparib treatment due to clinical disease progression after 32 weeks on study and subsequently received palliative radiotherapy due to painful bone lesions.

Following discontinuation from the rucaparib treatment, the patient received carboplatin and cabazitaxel for 2 cycles (intravenous infusion) until subsequent scans indicated progressive disease in nontarget liver lesions two months later. The patient discontinued carboplatin/cabazitaxel and did not receive any further anticancer therapies. The patient died ≈23 months after initial diagnosis due to progression of his disease.

## Discussion and Conclusions

Here we have reported on a patient with BRCA-mutated mCRPC who had a response to treatment with the PARP inhibitor rucaparib, remaining on rucaparib treatment for >2 times longer than each of the other therapies he received. We hypothesize the response of the patient’s tumor to rucaparib was likely driven by DDR deficiency caused by homozygous loss of all *BRCA2* exons. Notably, tissue NGS was conducted on an archival tumor sample obtained at the time of initial diagnosis, while plasma NGS was conducted on samples obtained prior to rucaparib treatment (43 weeks after diagnosis). The tissue contained a *BRCA1* missense alteration with a computationally inferred deleterious effect, while the patient’s plasma yielded a homozygous whole-gene *BRCA2* deletion in addition to the *BRCA1* alteration.

This result demonstrates the challenges of selecting the “right” sample and assay type as well as the right time for molecular profiling. Since both Oncomine and FoundationOne Liquid CDx assays are capable of detecting all alteration types (19, 20), a possible explanation for the discordance is temporal and/or spatial heterogeneity between tumor deposits. For example, the retroperitoneal lymph node (which did not meet size criteria for tracking as a malignant lesion, after biopsy) may not have contained the *BRCA2* homozygous loss (yet), whereas the metastases, such as those in the liver (which did respond) and bone, may have contained this alteration. This highlights the ability of plasma testing to overcome the limitation of sampling a single lesion because plasma may contain DNA from both primary and metastatic lesions (15), provided that sufficient tumor content is present in the plasma, which may not be the case for patients with low tumor burden or who are responding to a therapy. Alternatively, it is possible that the *BRCA2* homozygous loss was acquired subsequent to the initial lymph node biopsy after docetaxel and abiraterone treatment. Somatic BRCA alterations that arise during the course of disease progression and/or treatment have been shown to make up half of all BRCA alterations in mCRPC (21), among them homozygous BRCA loss, which accounts for roughly 20% of all BRCA alterations in mCRPC (22). Therefore, another advantage of plasma testing is its ability to query the current genomic landscape rather than that of an earlier disease stage when tissue biopsies are typically performed.

In a recent analysis, >90% of patients with mCRPC had detectable circulating tumor DNA (ctDNA) (23), and the frequency of alterations detected in ctDNA was similar to that reported in studies of tissue biopsies (21, 24, 25). Further, the high concordance (75–90%) between matched plasma and tissue pairs in analyses from TRITON2 (26) and the phase 3 PROfound study of olaparib (27) highlights the utility of plasma testing in detecting alterations in genes of interest, such as *BRCA1* and *BRCA2.* However, it is interesting to note cases such as the current patient, where discordant results have identified additional actionable gene alterations. Importantly, the objective and PSA response rates following rucaparib treatment were similar in TRITON2 patients with mCRPC who had BRCA alterations detected by tissue or by plasma (26).

In general, genomic analysis of cfDNA purified from blood can be a practical alternative to tumor tissue testing for patients with mCRPC (28, 29). Patients with mCRPC rarely undergo routine biopsy sampling, and archival biopsy tissue collected from a single site at diagnosis may be less representative of the metastatic disease state (30, 31). As mCRPC largely targets the bones, many patients lack accessible soft tissue lesions for a contemporaneous biopsy (14). However, cfDNA can be obtained from patients through a minimally invasive blood draw to evaluate the contemporaneous genomic tumor landscape. An important concern in plasma testing is potential false-positive results from the cfDNA assay due to technical or biological factors, such as low variant allele frequency and clonal hematopoiesis of indeterminate potential (CHIP); therefore, use of plasma assays may be complemented by sequencing of tissue and/or matched nontumor samples (32–36). Technical and analytical methods have been developed to address difficulties such as low levels of tumor DNA (37) and detecting certain types of genomic changes (eg, fusions, deletions, copy number variations) (38, 39). Simultaneously, missing patients who may be eligible for targeted treatment may be a real concern when sequencing a single sample to obtain a genomic snapshot at one specific timepoint. Testing multiple samples from a patient over the course of his disease to capture the genomic topography as it evolves may be advisable.

In summary, this case is an example highlighting several advantages of plasma testing compared with tissue testing when selecting targeted therapies for treatment of mCRPC. However, in all cases, physicians will have to determine which tool presents the best solution for any given patient’s clinical circumstances.

## Data Availability Statement

The original contributions presented in the study are included in the article. Further inquiries can be directed to the corresponding author.

## Ethics Statement

The TRITON2 study involved human participants, was reviewed and approved by national or local institutional review boards including Yale IRB (protocol ID 2000020433), and was performed in accordance with the Declaration of Helsinki and Good Clinical Practice guidelines of the International Council for Harmonisation. The patients/ participants provided their written informed consent to participate in this study. Written informed consent was obtained from the individual(s) for the publication of any potentially identifiable images or data included in this article.

## Author Contributions

SW and AL were involved in the TRITON2 study design. DP, SW, and AL collected the data, analyzed and interpreted the data, drafted and revised the manuscript, and read and approved the final manuscript. All authors attest to accountability for the accuracy and integrity of the manuscript.

## Funding

TRITON2 was funded by Clovis Oncology, Inc.

## Conflict of Interest

DP reports consulting fees for Clovis Oncology, Ada Cap (Advanced Accelerator Applications), Amgen, Astellas, AstraZeneca, Bayer, Bicycle Therapeutics, Boehringer Ingelheim, Bristol Myers Squibb, Eli Lilly, Exelixis, Gilead Sciences, Incyte, Ipsen, Janssen, Mirati, Monopteros, Pfizer, Pharmacyclics, Regeneron, Roche, Seattle Genetics, and Urogen, grant support from Clovis Oncology, Ada Cap (Advanced Accelerator Applications), Agensys Inc, Astellas, AstraZeneca, Bayer, BioXcel Therapeutics, Bristol Myers Squibb, Eisai, Eli Lilly, Endocyte, Genentech, Gilead Sciences, Innocrin, MedImmune, Medivation, Merck, Mirati, Novartis, Pfizer, Progenics, Replimune, Roche, Sanofi Aventis, and Seattle Genetics, and prior ownership interest/investment in Bellicum (sold 7/2020), Tyme (sold 10/2019). SW and AL are employees of Clovis Oncology and may own stock or have stock options in that company.

## Publisher’s Note

All claims expressed in this article are solely those of the authors and do not necessarily represent those of their affiliated organizations, or those of the publisher, the editors and the reviewers. Any product that may be evaluated in this article, or claim that may be made by its manufacturer, is not guaranteed or endorsed by the publisher.

## References

[1] Leongamornlert D Mahmud N Tymrakiewicz M Saunders E Dadaev T Castro E. Germline BRCA1 Mutations Increase Prostate Cancer Risk. Br J Cancer (2012) 106:1697–701. doi: 10.1038/bjc.2012.146 PMC3349179 22516946

[2] Thompson D Easton DF. Cancer Incidence in BRCA1 Mutation Carriers. J Natl Cancer Inst (2002) 94:1358–65. doi: 10.1093/jnci/94.18.1358 12237281

[3] Kote-Jarai Z Leongamornlert D Saunders E Tymrakiewicz M Castro E Mahmud N. BRCA2 is a Moderate Penetrance Gene Contributing to Young-Onset Prostate Cancer: Implications for Genetic Testing in Prostate Cancer Patients. Br J Cancer (2011) 105:1230–4. doi: 10.1038/bjc.2011.383 PMC3208504 21952622

[4] van Asperen CJ Brohet RM Meijers-Heijboer EJ Hoogerbrugge N Verhoef S Vasen HFA. Cancer Risks in BRCA2 Families: Estimates for Sites Other Than Breast and Ovary. J Med Genet (2005) 42:711–9. doi: 10.1136/jmg.2004.028829 PMC1736136 16141007

[5] Rubraca (Rucaparib) Tablets [Prescribing Information]. Boulder, CO: Clovis Oncology, Inc (2021).

[6] Lynparza (Olaparib) Tablets [Prescribing Information]. Wilmington: DE: AstraZeneca Pharmaceuticals (2021).

[7] Abida W Patnaik A Campbell D Shapiro J Bryce AH McDermott R. Rucaparib in Men With Metastatic Castration-Resistant Prostate Cancer Harboring a *BRCA1* or *BRCA2* Gene Alteration. J Clin Oncol (2020) 38:3763–72. doi: 10.1200/JCO.20.01035 PMC7655021 32795228

[8] de Bono J Mateo J Fizazi K Saad F Shore N Sandhu S. Olaparib for Metastatic Castration-Resistant Prostate Cancer. N Engl J Med (2020) 382:2091–102. doi: 10.1056/NEJMoa1911440 32343890

[9] Messina C Cattrini C Soldato D Vallome G Caffo O Castro E. BRCA Mutations in Prostate Cancer: Prognostic and Predictive Implications. J Oncol (2020) 2020:4986365. doi: 10.1155/2020/4986365 32963528 PMC7492871

[10] NCCN Clinical Practice Guidelines in Oncology. Prostate Cancer (Version 4.2022) National Comprehensive Cancer Network (NCCN) (2022). Available at: https://www.nccn.org/professionals/physician_gls/pdf/prostate.pdf (Accessed May 11, 2022).

[11] Gillessen S Attard G Beer TM Beltran H Bjartell A Bossi A. Management of Patients Wth Advanced Prostate Cancer: Report of the Advanced Prostate Cancer Consensus Conference 2019. Eur Urol (2020) 77:508–47. doi: 10.1016/j.eururo.2020.01.012 32001144

[12] Giri VN Knudsen KE Kelly WK Cheng HH Cooney KA Cookson MS. Implementation of Germline Testing for Prostate Cancer: Philadelphia Prostate Cancer Consensus Conference 2019. J Clin Oncol (2020) 38:2798–811. doi: 10.1200/JCO.20.00046 PMC7430215 32516092

[13] Pujol P Barberis M Beer P Friedman E Piulats JM Capoluongo ED. Clinical Practice Guidelines for BRCA1 and BRCA2 Genetic Testing. Eur J Cancer (2021) 146:30–47. doi: 10.1016/j.ejca.2020.12.023 33578357

[14] Ross JS Ali SM Wang K Palmer G Yelensky R Lipson D. Comprehensive Genomic Profiling of Epithelial Ovarian Cancer by Next Generation Sequencing-Based Diagnostic Assay Reveals New Routes to Targeted Therapies. Gynecol Oncol (2013) 130:554–9. doi: 10.1016/j.ygyno.2013.06.019 23791828

[15] Oliveira KCS Ramos IB Silva JMC Barra WF Riggins GJ Palande V. Current Perspectives on Circulating Tumor DNA, Precision Medicine, and Personalized Clinical Management of Cancer. Mol Cancer Res (2020) 18:517–28. doi: 10.1158/1541-7786.MCR-19-0768 31996469

[16] Brierley JD Gospodarowicz MK Wittekind C. (eds) TNM Classification of Malignant Tumours. Eighth ed. Chichester, West Sussex, UK: John Wiley & Sons, Ltd (2017).

[17] Amin MB Edge SB Greene FL Byrd DR Brookland RK Washington MK. (eds) AJCC Cancer Staging Manual. 8th ed. New York, NY, USA: Springer (2017).

[18] Landrum MJ Lee JM Benson M Brown GR Chao C Chitipiralla S. ClinVar: Improving Access to Variant Interpretations and Supporting Evidence. Nucleic Acids Res (2018) 46:D1062–D7. doi: 10.1093/nar/gkx1153 PMC5753237 29165669

[19] Dehghani M Rosenblatt KP Li L Rakhade M Amato RJ. Validation and Clinical Applications of a Comprehensive Next Generation Sequencing System for Molecular Characterization of Solid Cancer Tissues. Front Mol Biosci (2019) 6:82. doi: 10.3389/fmolb.2019.00082 31681791 PMC6798036

[20] Clark TA Chung JH Kennedy M Hughes JD Chennagiri N Lieber DS. Analytical Validation of a Hybrid Capture–Based Next-Generation Sequencing Clinical Assay for Genomic Profiling of Cell-Free Circulating Tumor DNA. J Mol Diagn (2018) 20:686–702. doi: 10.1016/j.jmoldx.2018.05.004 29936259 PMC6593250

[21] Pritchard CC Mateo J Walsh MF De Sarkar N Abida W Beltran H. Inherited DNA-Repair Gene Mutations in Men With Metastatic Prostate Cancer. N Engl J Med (2016) 375:443–53. doi: 10.1056/NEJMoa1603144 PMC4986616 27433846

[22] Abida W Campbell D Patnaik A Sautois B Shapiro JD Vogelzang NJ. Genomic Characteristics Associated With Clinical Activity of Rucaparib in Patients (Pts) With BRCA1 or BRCA2 (BRCA)-Mutated Metastatic Castration-Resistant Prostate Cancer (mCRPC). J Clin Oncol (2020) 38:abst 178. doi: 10.1200/JCO.2020.38.6_suppl.178 PMC7655021 32795228

[23] Tukachinsky H Madison RW Chung JH Gjoerup OV Severson EA Dennis L. Genomic Analysis of Circulating Tumor DNA in 3,334 Patients With Advanced Prostate Cancer Identifies Targetable BRCA Alterations and AR Resistance Mechanisms. Clin Cancer Res (2021) 27:3094–105. doi: 10.1158/1078-0432.CCR-20-4805 PMC9295199 33558422

[24] Abida W Armenia J Gopalan A Brennan R Walsh M Barron D. Prospective Genomic Profiling of Prostate Cancer Across Disease States Reveals Germline and Somatic Alterations That May Affect Clinical Decision Making. JCO Precis Oncol (2017) 1:1–16. doi: 10.1200/po.17.00029 PMC5558263 28825054

[25] Robinson D Van Allen Eliezer M Wu Y-M Schultz N Lonigro Robert J Mosquera J-M. Integrative Clinical Genomics of Advanced Prostate Cancer. Cell (2015) 161:1215–28. doi: 10.1016/j.cell.2015.05.001 PMC4484602 26000489

[26] Abida W Patnaik A Campbell D Shapiro J Bryce AH McDermott R. Clinical Activity of Rucaparib in Patients With Metastatic Castration-Resistant Prostate Cancer (mCRPC) and BRCA1 or BRCA2 Mutations Identified by FoundationOne® Liquid CDx (F1L CDx). Prostate Cancer Foundation (2020). doi: 10.26226/morressier.5f69edb69b74b699bf38c657

[27] Chi KN Barnicle A Sibilla C Lai Z Corcoran C Williams JA. Concordance of BRCA1, BRCA2 (BRCA), and ATM Mutations Identified in Matched Tumor Tissue and Circulating Tumor DNA (ctDNA) in Men With Metastatic Castration-Resistant Prostate Cancer (mCRPC) Screened in the PROfound Study. J Clin Oncol (2021) 39(suppl 6):abst 26. doi: 10.1200/JCO.2021.39.6_suppl.26

[28] Hegemann M Stenzl A Bedke J Chi KN Black PC Todenhöfer T. Liquid Biopsy: Ready to Guide Therapy in Advanced Prostate Cancer? BJU Int (2016) 118:855–63. doi: 10.1111/bju.13586 27430478

[29] Frenel JS Carreira S Goodall J Roda D Perez-Lopez R Tunariu N. Serial Next-Generation Sequencing of Circulating Cell-Free DNA Evaluating Tumor Clone Response to Molecularly Targeted Drug Administration. Clin Cancer Res (2015) 21:4586–96. doi: 10.1158/1078-0432.CCR-15-0584 PMC4580992 26085511

[30] Hovelson DH Tomlins SA. The Role of Next-Generation Sequencing in Castration Resistant Prostate Cancer Treatment. Cancer J (2016) 22:357–61. doi: 10.1097/PPO.0000000000000217 PMC5759046 27749331

[31] Beltran H Rubin MA. New Strategies in Prostate Cancer: Translating Genomics Into the Clinic. Clin Cancer Res (2013) 19:517–23. doi: 10.1158/1078-0432.ccr-12-1452 PMC4123124 23248095

[32] Bowman RL Busque L Levine RL. Clonal Hematopoiesis and Evolution to Hematopoietic Malignancies. Cell Stem Cell (2018) 22:157–70. doi: 10.1016/j.stem.2018.01.011 PMC5804896 29395053

[33] Stetson D Ahmed A Xu X Nuttall BRB Lubinski TJ Johnson JH. Orthogonal Comparison of Four Plasma NGS Tests With Tumor Suggests Technical Factors are a Major Source of Assay Discordance. JCO Precis Oncol (2019) 3:1–9. doi: 10.1200/po.18.00191 35100678

[34] Jensen K Konnick EQ Schweizer MT Sokolova AO Grivas P Cheng HH. Association of Clonal Hematopoiesis in DNA Repair Genes With Prostate Cancer Plasma Cell-Free DNA Testing Interference. JAMA Oncol (2021) 7:107–10. doi: 10.1001/jamaoncol.2020.5161%JJAMAOncology PMC7645740 33151258

[35] Beware Liquid Biopsies to Guide PARP Blockade. Cancer Discovery (2021) 11:6. doi: 10.1158/2159-8290.Cd-nb2020-105 33208392

[36] Reichert ZR Jones MA Alumkal JJ. A CHIP in the Armor of Cell-Free DNA–based Predictive Biomarkers for Prostate Cancer. JAMA Oncol (2021) 7:111–2. doi: 10.1001/jamaoncol.2020.5140%JJAMAOncology 33151257

[37] Lu YT Delijani K Mecum A Goldkorn A. Current Status of Liquid Biopsies for the Detection and Management of Prostate Cancer. Cancer Manag Res (2019) 11:5271–91. doi: 10.2147/CMAR.S170380 PMC6559244 31239778

[38] Huang C-C Du M Wang L. Bioinformatics Analysis for Circulating Cell-Free DNA in Cancer. Cancers (2019) 11:805. doi: 10.3390/cancers11060805 PMC6627444 31212602

[39] Keller L Belloum Y Wikman H Pantel K. Clinical Relevance of Blood-Based ctDNA Analysis: Mutation Detection and Beyond. Br J Cancer (2021) 124:345–58. doi: 10.1038/s41416-020-01047-5 PMC7852556 32968207

